# A Modified Monomeric Red Fluorescent Protein Reporter for Assessing CRISPR Activity

**DOI:** 10.3389/fcell.2018.00054

**Published:** 2018-05-15

**Authors:** Camilla Højland Knudsen, Emilía S. Ásgrímsdóttir, Karim Rahimi, Katherine P. Gill, Søs Frandsen, Susanne Hvolbøl Buchholdt, Muwan Chen, Jørgen Kjems, Fabia Febbraro, Mark Denham

**Affiliations:** ^1^Danish Research Institute of Translational Neuroscience - DANDRITE, Nordic-EMBL Partnership for Molecular Medicine, Aarhus University, Aarhus, Denmark; ^2^Department of Biomedicine, Aarhus University, Aarhus, Denmark; ^3^Department of Molecular Biology and Genetics, Aarhus University, Aarhus, Denmark; ^4^Interdisciplinary Nanoscience Center, Aarhus University, Aarhus, Denmark; ^5^Department of Health Science and Technology, Aalborg University, Aalborg, Denmark

**Keywords:** CRISPR/Cas9, pluripotent stem cells, INDEL mutation, homologous recombination, human embryonic stem cells, gene targeting techniques, safe harbor loci

## Abstract

Gene editing in human embryonic stem cells (hESCs) has been significantly enhanced by the discovery and development of CRISPR Cas9, a programmable nuclease system that can introduce targeted double-stranded breaks. The system relies on the optimal selection of a sgRNA sequence with low off-targets and high efficiency. We designed an improved monomeric red fluorescent protein reporter, GEmCherry2, for assessing CRISPR Cas9 activity and for optimizing sgRNA. By incorporating an out-of-frame sequence to the N-terminal of the red fluorescent protein mCherry, we created a visual tool for assessing the indel frequency after cutting with CRISPR Cas9. When a sgRNA-Cas9 construct is co-transfected with a corresponding GEmCherry2 construct, single nucleotide indels can move the GEmCherry2 sequence back in-frame and allow quantification and comparison of the efficiency of different sgRNA target sites by measuring red fluorescence. With this GEmCherry2 assay, we compared four target sites in the safe harbor *AAVS1* locus and found significant differences in target site activity. We verified the activity using TIDE, which ranked our target sites in a similar order as the GEmCherry2 system. We also identified an AAV short inverted terminal repeat sequence within the Cas9 construct that, upon removal significantly improved transient transfection and expression in hESCs. Moreover, using GEmCherry2, we designed a sgRNA to target *SORCS2* in hESCs and successfully introduced indels into the coding sequence of *SORCS2*.

## Introduction

Human embryonic stem cells (hESCs) and human induced pluripotent stem cells (hiPSCs) are an invaluable cell source for recapitulating and studying early developmental or disease processes. Furthermore, refined differentiation methods capable of generating patient-specific cell derivatives have the potential to improve cell replacement therapies for numerous diseases, including neurodegenerative diseases such as Parkinson's disease. In addition to hiPSC technology, recent advancements in gene editing techniques have aided these efforts by increasing the ease at which genome editing can be performed, for applications including correction or deletion of genes or creation of reporter cell lines for the study of developmental processes.

During the last decade, several gene-editing techniques have been developed, such as Zinc finger nucleases (ZFN) and the transcription activator-like effector nucleases (TALENS) which feature a customizable DNA binding domain, allowing its protein sequence to be modified to target specific genomic sequences. The requirements to custom-modify the protein sequence for each target site limits its broad application. Alternatively, the clustered regularly interspersed palindromic repeats (CRISPR) associated nuclease Cas9 system can be modified to target specific DNA sites; this system is simple to customize as it uses the same protein sequence in each case. The type II CRISPR Cas9 system requires only the modification of a synthetic single guide RNA (sgRNA), which can be programmed to identify a 20 base-pair sequence that is followed by a protospacer-adjacent motif (PAM) sequence -NGG. This sgRNA modification is therefore inexpensive and straightforward in comparison to ZFN and TALENS. Furthermore, this system results in a site-specific double-stranded break (DSB) between the 17th and 18th bases in the target sequence, unlike the indiscriminate cutting employed by the Fokl endonuclease used in ZNF and TALENS. The CRISPR system is therefore easily programmed and results in a site-specific DSB, making it a valuable tool for iPSC genome engineering.

Previous reports have shown that the guide RNA architecture and targeting site can both influence Cas9 activity (Sander and Joung, 2014). Several methods for assessing Cas9 activity has been reported, including the use of a surrogate dual fluorescent reporter system, SURVEYOR, RFLP, TIDE, whole genome, or exome sequencing (Kim et al., 2011; Ran et al., 2013b; Brinkman et al., 2014; Cho et al., 2014; Kuhar et al., 2016; Zhou et al., 2016). Although all these systems can determine Cas9 activity, some also capable of assessing off-targets, they are somewhat hampered by either low sensitivity, labor-intensive procedures, or use multiple fluorescent proteins that prevent the assessment of Cas9 co-transfection. Here we describe the development of a modified monomeric red fluorescent protein (RFP) for measuring Cas9 activity, which serves as a rapid tool for evaluating sites with high Cas9 activity. We demonstrate that this technique can be rapidly employed to identify highly efficient sites within the *AAVS1* safe harbor locus or in a protein coding sequence. Furthermore, for efficient plasmid transfection of Cas9 sgRNA constructs into hPSCs we increased transient expression by removal of an Adeno-associated virus (AAV) inverted terminal repeat (ITR) sequence that inhibited transgene expression.

## Methods

### hESC culture

The H9 (WA-09, WiCell) hESC line was cultured as previously described (Denham and Dottori, 2011). Briefly, cells were maintained on a feeder layer of mitomycin-C treated human foreskin fibroblasts in KSR media (DMEM/F-12, 0.1 mM β-mercaptoethanol, 1% nonessential amino acids, 2 mM glutamine, 0.5% pen/strep, 20% knockout serum replacement) containing 15 ng/mL Activin A and 15 ng/mL FGF2. Before Nucleofection or infection, H9 was transferred to a feeder-free system on Vitronectin coated plates in E8 media (StemCell Technologies). All cells were grown at 37°C and 5% CO_2_.

### HEK293T culture

HEK293T cells were cultured in FDMEM (DMEM 4.5%glucose, 10% FBS, 0.5% pen/strep, 1% Glutamax, 1% non-essential amino acids, 1% sodium pyruvate; ThermoFisher Scientific) in T75 (SARSTEDT) flasks and split by trypsination before reaching confluency. Cells were grown at 37°C, 5% CO_2_.

### Lipofectamine transfection of HEK293T cells

HEK293T cells were transfected using Lipofectamine 2000 according to manufacturer's protocol. On the day before transfection, the cells were plated in multi-well plates (6-well or 12-well (SARSTEDT)) to reach ~85% confluency at the time of transfection. The cells were transfected with up to 2 μg of total plasmid DNA per well for 6-well plates and up to 1 μg of total plasmid DNA per well for 12-well plates.

### Nucleofection of hESCs

H9 cells from Vitronectin plates were dissociated to single cells using Accutase (ThermoFisher Scientific) after being incubated with 10 μM ROCK inhibitor (Y27632) for at least 1 h. After diluting the Accutase with E8 media, the cells were counted and 200,000 cells per reaction were spun down (300 xG, 5 min) and resuspended in premixed Nucleofection solution, P3 (Lonza). Twenty micro-liters of cell suspension was added to a maximum of 2 μg of total DNA (no more than 2 μL) and transferred to a Nucleofection chamber (16-well strip). The H9 cells were transfected using parameter CM 130, P3 solution, and plated out on Vitronectin coated plates (35 mm, Nunc) in E8 media supplemented with 10 μM ROCK inhibitor. Media was changed to regular E8 media after 1–2 days.

### Fluorescence activated cell sorting (FACS) analysis

HEK293T cells were co-transfected with GEmCherry and corresponding CRISPR-Cas9 vectors. The cells were imaged under a fluorescent microscope at 48 h post-transfection. After imaging, the cells were trypsinised (0.25% trypsin-EDTA) to a single cell suspension, washed in phosphate buffered saline (PBS) and fixed in 4% paraformaldehyde (PFA) for 20 min at 4°C. The fixed cells were washed and put through a 40 μm cell strainer before being sorted against GFP and mCherry on an LSR Fortessa cell analyser.

### Polymerase chain reactions (PCRs)

PCR of genomic *AAVS1* regions was carried out with OneTaq hot start DNA polymerase (NEB) as described by the manufacturer, with an initial denaturation step of 4 min and an annealing temperature of 63°C. Primers are listed in (Figure S1).

### Cloning

GEmCherry constructs were produced using the pmCerulean3-N1 (Addgene: 54730) backbone and cloning in the mCherry gene by PCR amplification from E[beta]C (Addgene: 24312) and N-terminal changes added by primer extension, producing the following vectors: pGEmCherry1, pGEmCherry2, pGEmCherry3, pGEmCherry4. The CBh promoter in pSpCas9(BB)-2A-GFP (PX458) (Addgene: 48138) was swapped for the CAG promoter, which was amplified by PCR from pPB-CAG.OSKM-puDtk (Yusa et al., 2009) producing the final vector pCAG-SpCas9-2A-GFP. The GEmCherry vector was cut with XbaI and BamHI (NEB) to allow insertion of target sites. The oligos (target sequences in Figure S1) were annealed before ligating them into the backbone finally producing GEmCherry2-C2, GEmCherry2-C3, GEmCherry2-C4, GEmCherry2-T2, GEmCherry2-TS1+3, and GEmCherry2-TS2. The Cas9 vector was constructed according to protocol (Ran et al., 2013b), producing pSpCas9-2A-GFP-sgC2, pSpCas9-2A-GFP-sgC3, pSpCas9-2A-GFP-sgC4, pSpCas9-2A-GFP-sgT2, pSpCas9-2A-GFP-sgSORCS2-G1, pSpCas9-2A-GFP-sgSORCS2-G2, pSpCas9-2A-GFP-sgSORCS2-G3, and LentiCRISPRv2-sgSORCS2-G2. Cloning was carried out using electrocompetent Stbl3 or Top10 *E. coli* cells. The AAV ITR fragment was removed from pCAG-SpCas9-2A-GFP by double digestion with NotI and SbfI to remove a 145 bp fragment including the ITR element. After removing the ITR element, two ssDNA oligos were synthesized (Sigma) and annealed to produce a fragment containing a BamHI site with two sticky ends compatible with the NotI and SbfI and ligated in with T4 DNA ligase kit (ThermoFisher EL0016) according to the manufacturer instructions. The following vector without an ITR was produced: pCAG-SpCas9-2A-GFP-noITR. All plasmids have been made publicly available through the Addgene non-profit plasmid repository.

### Lentiviral production and infection

Lentivirus was produced similarly to what was previously described (Denham et al., 2010). HEK293T cells were transfected by Lipofectamine 2000 with plasmids LentiCRISPRv2_SORCS2-G2, envelope plasmid pMD2.G, and packaging vector psPAX2 to generate lentiviral particles. The viral supernatant was harvested at 48 and 72 h post-transfection and concentrated by ultracentrifugation. Single cells of H9 on Vitronectin coated 35 mm dishes (6,000 cells per dish) were infected with concentrated lentivirus. At 48 h post-infection, Puromycin was added for 5 days (0.125 μg/mL Puromycin; ThermoFisher).

### Immunostaining

Immunocytochemistry was performed on cells grown on coverslips (VWR, Ø: 13 mm) and fixed in 4% PFA. Samples were blocked for 1 h at room temperature with 5% donkey serum in PBS-T (PBS with 0.25% triton-X). Primary antibodies were applied in blocking solution at room temperature overnight (GFP, rabbit, abcam: ab290, 1:1000; POU5F1, mouse IgG2b, Santa Cruz: sc-5279, 1:100) and washed off, 3x 10 min, with PBS-T and samples blocked for 10 min before applying secondary antibody in block solution for 1 h at room temperature (AlexaFluor 488 donkey anti-rabbit, 1:1000; AlexaFluor 568 donkey anti-mouse IgG (H + L), 1:1000). The secondary antibodies were washed off 3x 10 min in PBS-T before applying DAPI (1:1000 in PBS) for 10 min at room temperature. Coverslips were washed 3x 5 min with PBS before mounted on microscope slides with PVA DABCO (Sigma Aldrich).

### Spectral imaging

Emission wavelength and intensity of GEmCherry1, 2, and 3 was measured using λ-scan with ZEISS Confocal LSM800 microscope. Intensity was normalized within each sample and then plotted against emission wavelength in PRISM GraphPad 6.

### Sequencing

Sanger sequencing was carried out by EurofinsGenomics in ValueRead or Mix2Seq tubes. Sequencing samples were prepared according to manufacturers guidelines but with 300 ng of PCR product per sample. Sequencing primers are described in (Figure S1).

### Statistical analysis

FACS analysis was performed on 50,000 events per replicate using FlowJo software. Statistical analysis, consisting of one-way ANOVAs, adjusting for multiple comparisons with Tukey's correction, and unpaired two-tailed *t*-test was done using PRISM GraphPad 6. Mean gray-scale value was found using FIJI ImageJ on images taken with Zeiss Apotome microscope (no apotome function). Quantification of GFP/POU5F1 positive cells was done by measuring pixel area using FIJI ImageJ on images from Olympus IX53 microscope.

### TIDE analysis

TIDE analysis was performed on the *AAVS1* locus (PCR primers listed in Figure S1) from cultures sorted for GFP positive cells using the tide.nki.nl tool (version 0.1; Brinkman et al., 2014) run in R. The alignment window was set manually from where the Sanger sequencing chromatogram looked reliable and until 10 bp before the break site (corresponding R function modified to fix alignment window; code is available via osf.io/97ef4). The decomposition window was set at 400 bp, starting 42 bp downstream of the break site. The indel size range was set at 38 bp and the *p*-value significance cut-off at 0.001.

The reported efficiency of sgRNAs is likely an underestimate due to primer design, which did not allow for comparative analysis of an indel size range larger than 38 bp.

## Results

### Development of a monomeric RFP reporter for measuring CRISPR activity

**Figure 1:**
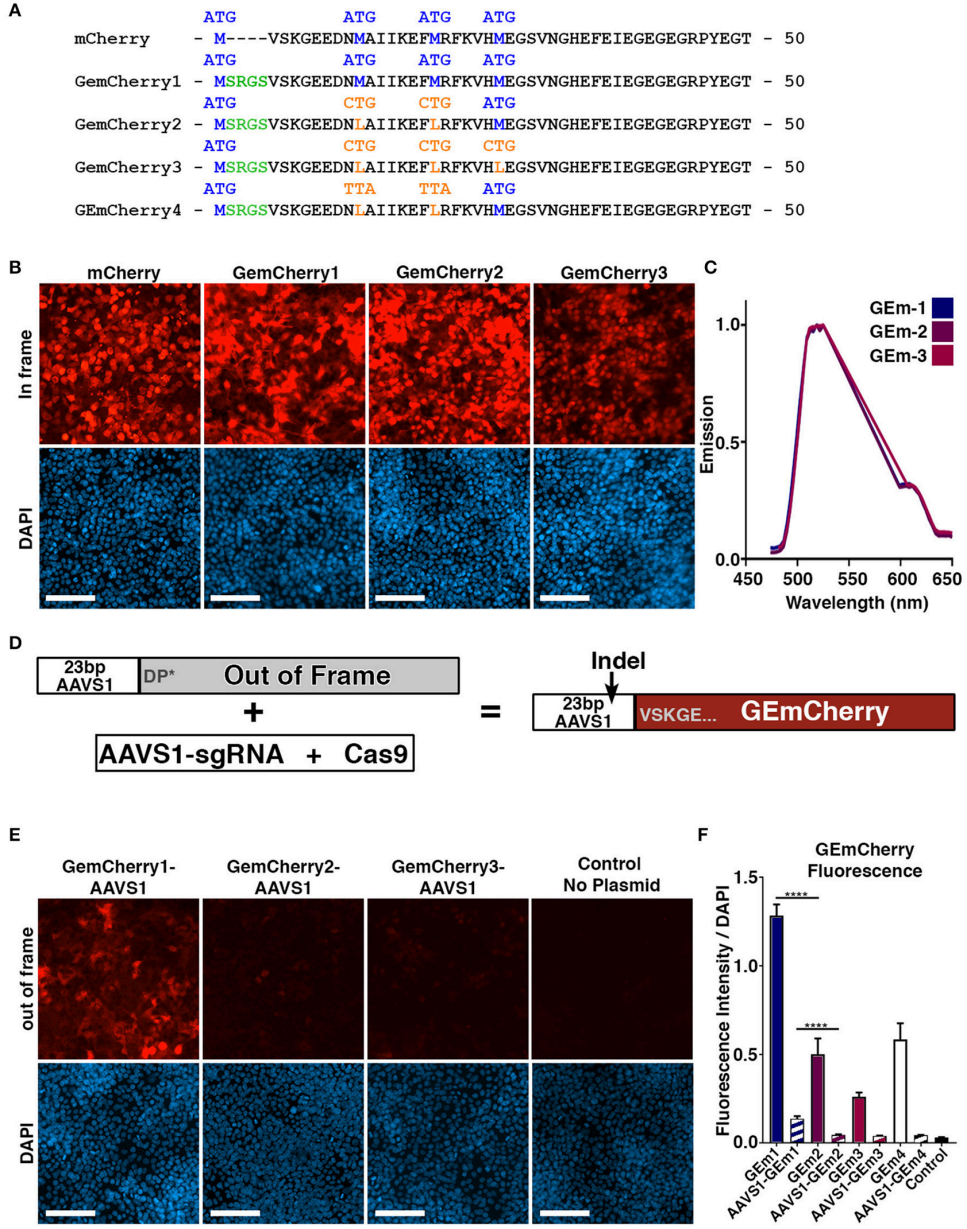
Development of monomeric RFP reporter for measuring CRISPR Cas9 activity. **(A)** Protein sequence of mCherry, GEmCherry1 with N-terminal modification to allow insertion of 23 bp target site, GEmCherry2 with two ATG → CTG mutations to decrease background fluorescence, GEmCherry3 with three ATG → CTG mutations and GEmCherry4 with two ATG → TTA mutations. **(B)** Images of HEK293T cells transfected with in-frame GEmCherry constructs (GEmCherry in red; DAPI in blue; scale bar = 100 μm). **(C)** λ-scan showing that only the intensity but not the emission spectra changes upon mutating the GEmCherry sequence. **(D)** By insertion of 23 bp target sequence, the reading frame will be pushed to induce a premature stop-codon abolishing fluorescence. Cas9 targeting and induction of indels will move the sequence back in-frame and again allow for the expression of red fluorescent protein. **(E)** HEK293T cells transfected with out-of-frame GEmCherry constructs (GEmCherry in red; DAPI in blue; scale bar = 100 μm). **(F)** Quantification of fluorescence intensity of in-frame (solid color) and out-of-frame (striped) constructs. In-frame intensity of GEmCherry1 was significantly higher than GEmCherry2, 3, and 4 (*p* < 0.0001, *n* = 3) but so was the out-of-frame background fluorescence (*p* < 0.0001, *n* = 3). The in-frame intensity between GEmCherry3 and GEmCherry2, and 4 was also significantly different (*p* = 0.0068 and *p* = 0.0008, *n* = 3) but the out-of-frame background fluorescence was not. For GEmCherry2, 3, and 4 the out-of-frame background fluorescence was not significantly different than the no-plasmid control, based on the pixel density, although low background fluorescence was visibly observable in both GEmCherry 2, 3, and 4. One-way ANOVA with Tukey's test were performed. Results are presented as mean with SD.

In order to obtain the highest targeting efficiency using CRISPR/Cas9 we first sought to develop a rapid system for evaluating sgRNA efficiency by developing a single fluorescent system to assess Cas9 activity that could be used in conjunction with existing CRISPR Cas9 GFP plasmids, such as the pCas9-GFP. This approach would allow for FACS based or visual assessment of double positive GFP/RFP fluorescence, resulting in a precise assessment of sgRNA efficiency. To achieve this, we incorporated an out-of-frame genomic target region into the N-terminal site of the mCherry fluorescent protein. Standard N-terminal fusion constructs maintain the original methionine start site and append N-terminal tags to the protein in-frame with an additional methionine. However, to facilitate short out-of-frame N-terminal sequences and prevent alternate start site initiation we removed the native methionine start site. This strategy was designed to prevent translation from the longest open reading frame when N-terminal fragments are out-of-frame. The append N-terminal fragment would correspond to the genomic fragment in this plasmid, which we named GEmCherry1 (Figures 1A,D).

Following transfection with GEmCherry1, and despite the methionine start site modifications, high fluorescence was observed when an out-of-frame sequence was inserted (Figure 1E). We reasoned that a downstream methionine site might be responsible for alternative initiation of translation. We identified that positions 14 and 21 contained methionine sites and modified these to leucine, an alternate amino acid with similar structure, generating the GEmCherry2 vector and additionally the 3rd ATG site at position 27 generating GEmCherry3 (Figure 1A). In addition, we generated a GEmCherry4 vector where CTG for leucine was mutated to TTA at positions 14 and 21, as we hypothesized that CTG could function as a weak alternative translation start site (Schwab et al., 2004). GEmCherry4 did not show significant differences in either in-frame or out-of-frame fluorescence compared to GEmCherry2 (Figure 1F). Transfection with in-frame sequences revealed that all modifications maintained red fluorescence (Figures 1B,C). Moreover, when an out-of-frame fragment was inserted, background fluorescence was significantly perturbed in GEmcherry2, GEmCherry3, and GEmCherry4 (Figure 1E). Only the intensity, and not the emission wavelength, changed upon mutating the GEmCherry sequence (Figure 1C).

Evaluation of all four constructs on background out-of-frame fluorescence and in-frame brightness revealed that GEmCherry2 was the superior choice with the lowest background fluorescence (67.89% lower than GEmCherry1, *p* < 0.0001) and maintained the brightest in-frame fluorescence (39.04% of GEmCherry1 fluorescence intensity, *p* < 0.0001; Figure 1F).

### Identification of an optimal CRISPR *AAVS1* site

**Figure 2:**
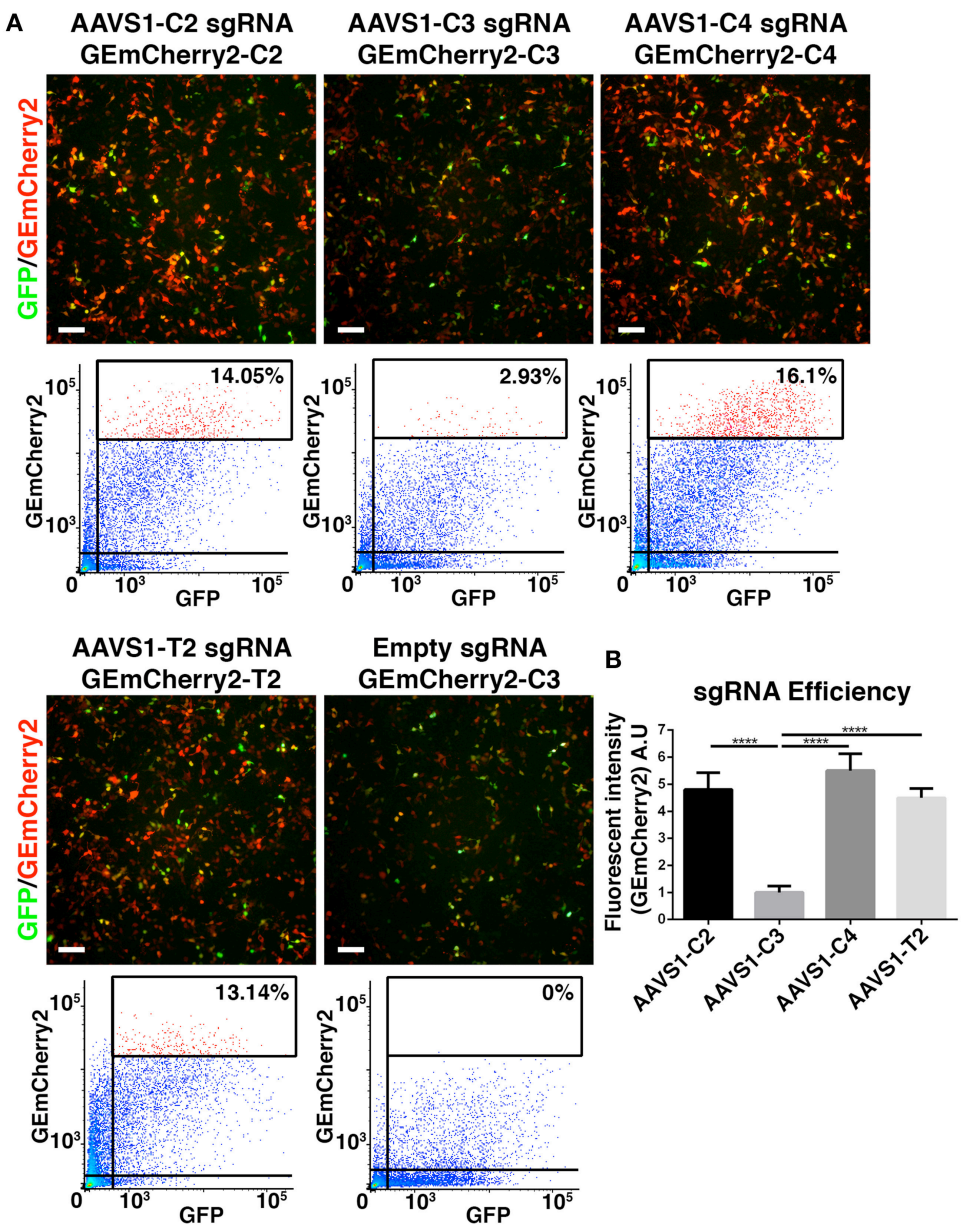
Identification of optimal CRISPR Cas9 *AAVS1* site. **(A)** HEK293T cells were co-transfected with CRISPR Cas9-GFP constructs with four different sgRNAs targeting the *AAVS1* locus and target site specific out-of-frame GEmCherry2 constructs (GEmCherry in red; GFP in green; scale bar = 100 μm). The shift to in-frame expression of RFP was visualized and the number of co-transfected, GFP+ and RFP+, cells were analyzed by FACS. **(B)** The fluorescent intensity, and thereby sgRNA efficiency, was estimated as high intensity RFP++ cells/GFP+ cells (percentages of RFP++ cells shown in FACS plots in **A)**. Cas9 activity at target site C2, C4, and T2 was, based on fluorescent intensity, estimated to be significantly higher (*p* < 0.0001, *n* = 3) than C3. We saw no significant difference in fluorescence intensity between C2, C4, and T2, although a trend was seen toward C4 being slightly more efficient (arbitrary unit). One-way ANOVA with Tukey's test was performed. Results are presented as mean with SD.

After successfully engineering a single fluorescent protein, GEmCherry2, which can facilitate an out-of-frame N-terminal fusion for measuring Cas9 activity, we tested whether GEmCherry2 could be used to measure Cas9 activity for a given genomic sequence. We chose the *AAVS1* locus (*PPP1R12C*) as a site, since it has been targeted successfully before with ZFN, TALENS, and CRISPR/Cas9 (Hockemeyer et al., 2009; Dekelver et al., 2010; Mali et al., 2013). Furthermore, this site is a known safe harbor locus, which makes it suitable for future transgene targeting. Firstly, we identified sequences with the lowest off-targets by CRISPOR (Haeussler et al., 2016). From this, we selected three sites designated C2, C3, and C4 (Figure S1) for assessing sgRNA Cas9 activity. These three sites were compared to the T2 site commonly used by others (Hockemeyer et al., 2009). Recognition sequences were cloned into GEmCherry2 and sgRNA sequences into the Cas9-GFP vector. Co-transfection and FACS analysis revealed that significant variation in Cas9 activity was observed between the genomic sequences (Figures 2A,B), confirming that sgRNA architecture and recognition site sequence contributes toward Cas9 activity. Based on GEmCherry2 fluorescence, we determined that sgRNA C4 had the highest efficiency, at 5.504 times that of C3 (*p* < 0.0001). C2 and T2 were also significantly greater than C3 [C2 (4.803), *p* < 0.0001; T2 (4.493), *p* < 0.0001]. No significant difference between C4, C2, and T2 were observed. Based on CRISPOR, C4 contained only two in-exon off-targets, both with four mismatches, whereas the T2 site contained 21 in-exon off-targets each with four mismatches and one in-exon off-target with a three base-pair mismatch (Figure S2). Overall, the off-target assessment and cutting efficiency of C4 makes it a superior choice when targeting the *AAVS1* locus with high Cas9 activity and the lowest potential off-targets.

### Comparative efficiency assessment of GEmCherry2 and TIDE assay

**Figure 3:**
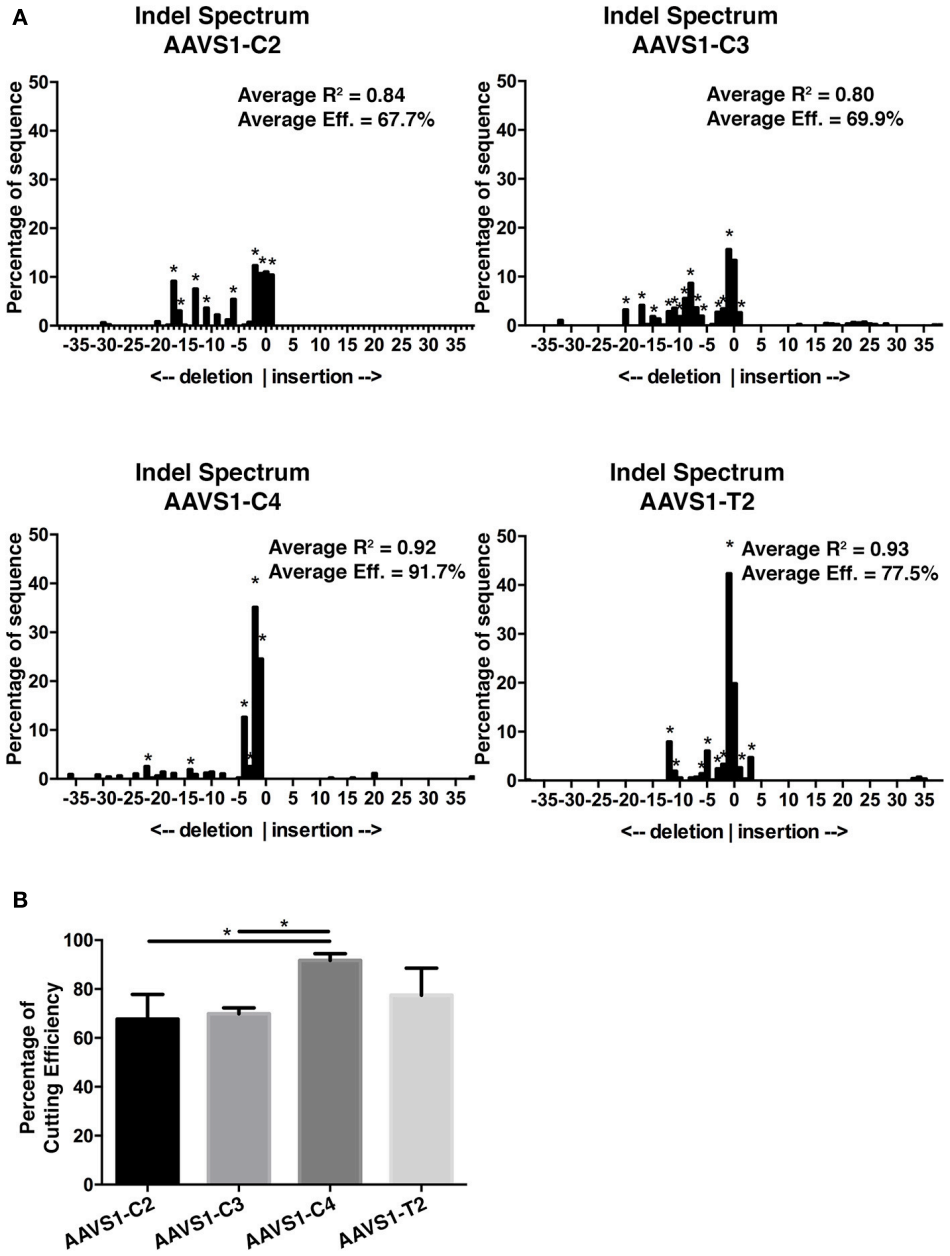
*AAVS1* sgRNA efficiency assessment by TIDE. **(A)** Representative indel spectrum analysis of *AAVS1* locus from HEK293T cells transfected with CRISPR Cas9-GFP sgRNA C2, C3, C4, or T2 (^*^p < 0.001; statistical analysis by TIDE version 0.1). The TIDE analysis was performed in three biological replicates. The average overall efficiency and the average *R*^2^ value for C2, C3, C4, and T2 is specified on each indel spectrum. **(B)** Overall CRISPR-Cas9 efficiency of C2, C3, C4, and T2 constructs as assessed by TIDE. C4 was significantly different from C2 and C3 (^*^p < 0.05, One-Way ANOVA with Tukey's test, *n* = 3, mean with SD). Cells were sorted for GFP prior to genomic DNA extraction.

Having determined that GEmCherry2 can be used to assess sgRNAs and Cas9 activity, we further sought to validate this technique and compare the efficiency determined by GEmCherry2 to Cas9 activity in the genomic locus. Epigenetic modifications can influence the ability of Cas9 to access the genomic DNA and may affect its activity in a genomic context. Therefore, we performed the TIDE assay (Brinkman et al., 2014) on the same CRISPR *AAVS1* target sites and calculated the indel frequency as a measure of Cas9 activity. The TIDE assay revealed that, similar to the GEmCherry2 assay, Cas9 activity varied between these sites (Figures 3A,B). Interestingly, the ranking of the various sites was in a similar order to that identified by GEmCherry2, with both methods identifying the AAVS1-C4 sgRNA as the most efficient (Figures 2B, 3A,B). This data reveals that GEmCherry2 can be effectively and rapidly used as a method for determining Cas9 activity.

### Gene editing in human embryonic stem cells

**Figure 4:**
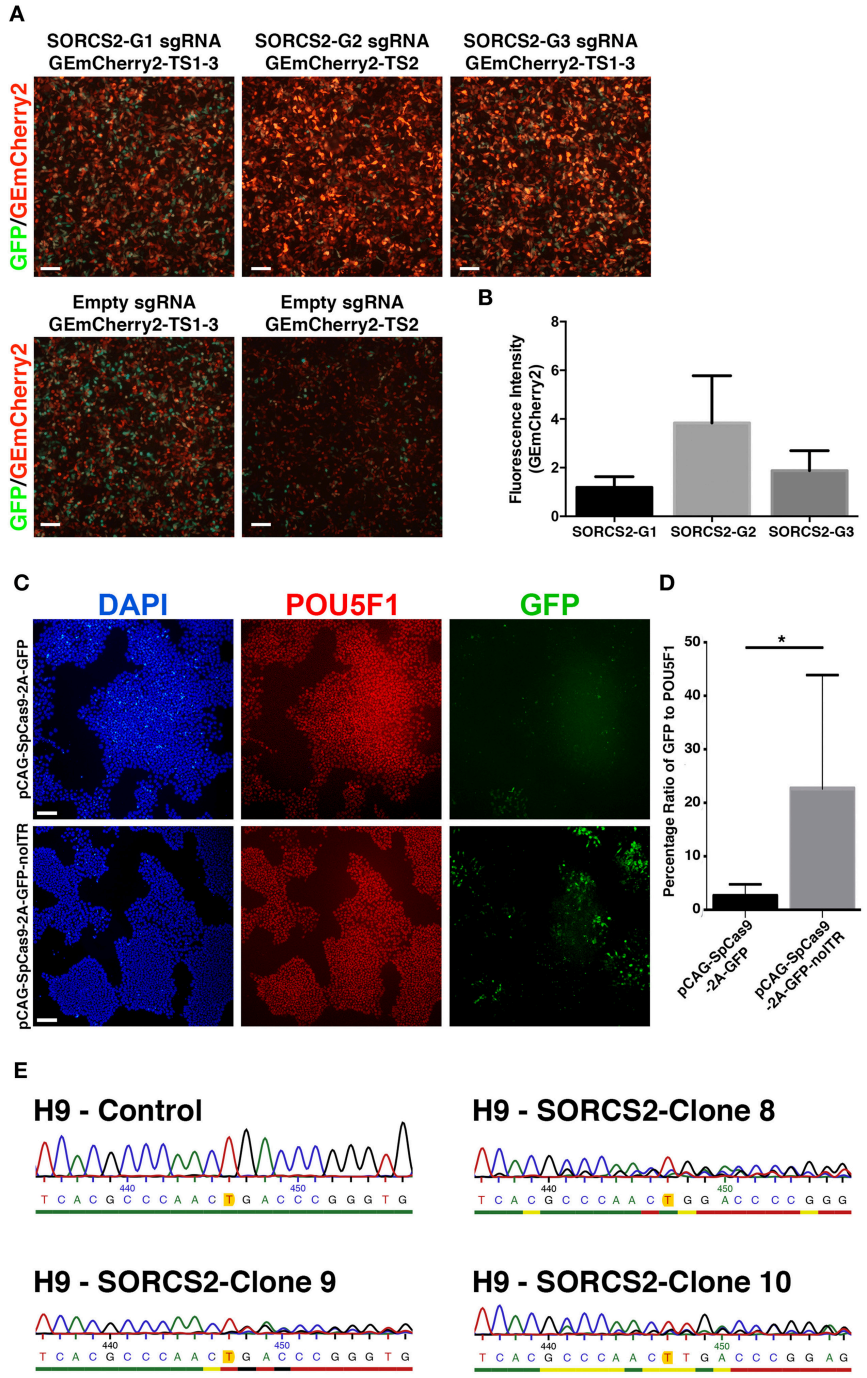
Gene editing in human embryonic stem cells. **(A)** Based on GEmCherry2 fluorescence we estimated the efficiency of three different sgRNAs (G1, G2, and G3) targeting exon1 of the *SORCS2* gene. HEK293T cells were co-transfected with CRISPR Cas9-GFP and GEmCherry2 constructs and the Cas9 cutting efficiency was estimated based on high-intensity in-frame fluorescence (GEmCherry in red; GFP in green; scale bar = 100 μm). **(B)** The fluorescence intensity (measured as pixel density) was quantified as GEmCherry against GFP fluorescence and normalized against the out-of-frame background fluorescence. The fluorescence intensity was not significantly different between the G1, G2, and G3 sgRNA, although a trend was seen toward G2 generating more high-intensity RFP++ cells (One-Way ANOVA with Tukey's test, *n* = 3, mean with SD). **(C)** Images of hESCs stained against POU5F1, GFP, and DAPI (scale bar = 100 μm) after transfection with either pCAG-SpCas9-2A-GFP (top) or pCAG-SpCas9-2A-GFP-noITR (bottom). **(D)** Quantification of transfection efficiency and GFP expression before and after deletion of the AAV ITR from the pCAG-SpCas9-2A-GFP vector (^*^p = 0.0446; *n*_ITR_ = 6, *n*_noITR_ = 5). Unpaired two-tailed *t*-test was performed. Results are presented as mean with *SD*. **(E)** Sanger sequencing of the genomic region of H9 control and three H9-SORCS2 knock-out cell lines (clone 8, 9, and 10). The expected cut site of sgRNA SORCS2-G2 is marked in yellow.

Finally, we used GEmCherry2 to design a sgRNA to knockout a protein-coding gene in hESCs. We chose to target *SORCS2*, a proneurotrophin receptor expressed in neurons and we designed three sgRNA against regions in the first coding exon (Glerup et al., 2014). Evaluating these with GEmCherry2, we identified sgRNA SORCS2-G2 as the most efficient sgRNA (Figures 4A,B). To introduce Cas9 and the sgRNA into hESCs, we tested the plasmid pSpCas9(BB)-2A-GFP (Ran et al., 2013b). After transfection, we found that transient expression from the Cas9 constructs [pSpCas9(BB)-2A-GFP (PX458); Addgene: 48138] was almost absent in comparison to other GFP reporter plasmids of similar size (data not shown). We therefore modified the plasmid and exchanged the Cbh promoter for a stronger CAG promoter, resulting in the plasmid pCAG-SpCas9-2A-GFP, which only resulted in marginal improvements in GFP expression (Figure 4C). After *in silico* analysis of the Cas9 plasmid sequence we identified an ITR sequence from an AAV within the construct as the only distinct difference between the plasmids. Subsequent removal of this AAV sequence was shown to significantly restore transfection efficiency and expression in hESCs (*p* = 0.0446; Figures 4C,D), resulting in the final plasmid pCAG-SpCas9-2A-GFP-noITR. In parallel to transient transfection, we produced lentiviral particles containing Cas9 and sgRNA SORCS2-G2. This method proved the most rapid and efficient targeting method in hESCs. From the lentiviral-infected hESCs, 10 clones were expanded and three analyzed for indels in *SORCS2* exon-1. Results showed that all three clones were successfully targeted and contained indels in *SORCS2* and, interestingly, clone 9 displayed a heterozygous sequence (Figure 4E).

## Discussion

Gene editing in human pluripotent stem cells is challenging in comparison to mouse pluripotent stem cells which can be clonally expanded in the presence of LIF, facilitating the survival and selection of targeted clones. Homology-directed repair methods have been improved significantly by the discovery of Cas9 and its development as a programmable nuclease system for targeted DSBs (Mali et al., 2013; Ran et al., 2013a). Despite these advancements, practical challenges of efficiently producing targeted clones remain. This study describes an optimized method for designing sgRNA sequences and an improved sgRNA delivery vector. Herein, we describe the development of a rapid assay system, GEmCherry2, which uses a single fluorescent reporter for assessing CRISPR Cas9 activity at sgRNA target sites. When compared with the TIDE assay, the GEmCherry2 assay showed a similar ranking of sequences (Brinkman et al., 2014). Additionally, and similar to the dual surrogate reporter system, the GEmCherry2 system can be used to assess ZFN, TALENS, DNA system efficiencies, or dual-nickase variants (Kim et al., 2011). Furthermore, an advantage of our reporter system over the dual fluorescent system is that it can be used in conjunction with a Cas9-GFP plasmid to evaluate Cas9 activity and overcome transfection efficiency discrepancies more precisely. Using this system as a primary screen, we were able to identify a novel *AAVS1* Cas9 active site with a low number of off-target sites and high Cas9 activity and we designed an efficient sgRNA for *SORCS2*. We attempted gene-targeting in hESCs and found that the majority of available CRISPR Cas9 plasmids contain an AAV ITR sequence in their backbone that reduces transfection efficiency and transient expression. This silencing may be occurring in hESCs through a similar mechanism to what has been reported in other cell types, by which the ITR regions are rapidly identified and methylated by the host cell (Chanda et al., 2017). After removal of these sequences, a significant improvement in transfection efficiency and transient expression was obtained. Furthermore, as an alternative and more efficient method for Cas9-mediated knock-out of SORCS2, we used lentiviruses, which are highly efficient at infecting hESCs (Gropp et al., 2003). With this rapid delivery system, we produced 10 clones and selected three for subsequent screening of indels in the *SORCS2* coding sequence. This approach proved to be highly efficient as all three clones showed indels in the region around the target site.

In addition to efficient sgRNA design, several methods exist for delivering Cas9 and sgRNA. The choice of delivery method will depend on the specific cell type and the application of either gene disruption or homologous recombination, which requires a repair template. Some of these methods include delivery of cell-penetrating Cas9 and guide RNA, capped RNA or integrase-deficient lentiviral vectors (Kim et al., 2014; Ramakrishna et al., 2014; Ortinski et al., 2017). In our approach, we used lentiviruses to infect hESCs. However, combining this with integrase-deficient or self-inactivating systems, such as Self-Limiting Cas9 circuit for Enhanced Safety and specificity (SLiCES) would be more desirable as they prevent the on-going expression of Cas9 (Petris et al., 2017).

Additional applications for GEmCherry2 also include assessing sgRNA activity against close matching off-target sequences in the genome or in patient lines with heterozygous single-nucleotide mutations. Sequences with multiple mismatches may be less problematic as modified version of Cas9 have recently been described, such as an enhanced Cas9 and a high-fidelity Cas9 that both have significantly reduced off-targets (Kleinstiver et al., 2016; Slaymaker et al., 2016). Nevertheless, the PAM-site restriction of spCas9 has also brought about the development and identification of alternate CRISPR endonucleases, which will all interact with off-targets differently (Hou et al., 2013; Kleinstiver et al., 2015; Zetsche et al., 2015; Hu et al., 2018).

In conclusion, we have developed a single fluorescent reporter system for evaluating sgRNA sequences and optimized a plasmid system for transfection into hPSCs. By combining the highest efficiency of genomic sgRNA sequence with improved plasmid transfection efficiency, a reduced number of cells would be required when performing homologous directed repair in hPSCs, which currently requires large-scale production to identify correctly targeted clones.

## Author contributions

MD: conceived and designed the experiments; CH, EÁ, KR, KG, SF, SH, MC, and FF: performed the experiments; JK: contributed instrumentation and expertise in the analysis; CH, and MD: analyzed data; MD and CH, wrote the manuscript. All authors reviewed the manuscript.

### Conflict of interest statement

The authors declare that the research was conducted in the absence of any commercial or financial relationships that could be construed as a potential conflict of interest. The reviewer GP and handling Editor declared their shared affiliation.

## Supplementary material

Figure S1 Primers and oligos used for targeting the *AAVS1* locus (*PPP1R12C*) and validating CRISPR Cas9 activity at the target sites. *AAVS1* target sites C2, C3, C4, and T2, including PAM-sequence, used for designing sgRNA and GEmCherry target sequences. Regional primers to PCR the *AAVS1* locus from genomic DNA from cells transfected with Cas9 constructs. Sequencing primers used for TIDE analysis, designed for sequencing to start close to the Cas9 cut site (65–120 bp upstream of cut site). *SORCS2* target sites G1, G2, and G3, including PAM-sequence (all in exon1). Regional primers to PCR the *SORCS2* G2 target site from genomic DNA from knock-out H9 cell lines. The G2 fwd primer was also used for Sanger sequencing.

Figure S2 Evaluation of PPP1R12C sgRNA design and off-target effects using http://crispor.tefor.net/ with input *PPP1R12C* sequence: *Homo sapiens (hg19)*, chr19:55626073-55628048, reverse genomic strand. The table shows the target sequence (guide sequence + PAM), specificity score, predicted efficiency, out-of-frame score, number of off-targets for up to four mismatches flanked by motif –NGG, –NAG, or –NGA (total/in-exon), and the number of mismatches and affected gene.
