# Supplementary material for: A Modified Monomeric Red Fluorescent Protein Reporter for Assessing CRISPR Activity
Source: Front Cell Dev Biol. 2018 May 15;6:54. doi: 10.3389/fcell.2018.00054 (PMC5962823; doi:10.3389/fcell.2018.00054)
Supplement: Figure S1 — Primers and oligos used for targeting the AAVS1 locus (PPP1R12C) and validating CRISPR Cas9 activity at the target sites. AAVS1 target sites C2, C3, C4, and T2, including PAM-sequence, used for designing sgRNA and GEmCherry target sequences. Regional primers to PCR the AAVS1 locus from genomic DNA from cells transfected with Cas9 constructs. Sequencing primers used for TIDE analysis, designed for sequencing to start close to the Cas9 cut site (65–120 bp upstream of cut site). SORCS2 target sites G1, G2, and G3, including PAM-sequence (all in exon1). Regional primers to PCR the SORCS2 G2 target site from genomic DNA from knock-out H9 cell lines. The G2 fwd primer was also used for Sanger sequencing. [file Data_Sheet_1.PDF]

Figure S1

PPP1R12C target sites:
C2: 5'- CCTTCTCCGACGGATGTCTCCCT **-** 3'
C3: 5'- CCTTCTCGGCGCTGCACCACGTG **-** 3'
C4: 5'- CCTGATATTGGGTCTAACCCCCA **-** 3'
T2: 5'- GGGGCCACTAGGGACAGGATTGG **-** 3'

PPP1R12C regional PCR primers:
Fwd primer: 5'- GAACTCTGCCCTCTAACGCT **-** 3'
Rev primer: 5'- GAAAGGCCCATTGCTCTCCT **-** 3'

Sequencing primers for TIDE analysis:
C2 seq primer: 5'- ATGGAGAGGTGGCTAAAGCCAG **-** 3'
C3 seq primer: 5'- TCTTCCTCCACTCCCTCTTC **-** 3'
C4 seq primer: 5'- CACTAGGGACAGGATTGGTGAC **-** 3'
T2 seq primer: 5'- CCGGTTAATGTGGCTCTGGTTC **-** 3'

SORCS2 target sites:
G1: 5'- GGGCCCTTCGAGGCGCGCGAGGG **-** 3'
G2: 5'- CCGCGGCACCCGGGTCAGTTGGG **-** 3'
G3: 5'- GGGCCCTCGCGCGCCTCGAAGGG **-** 3'

SORCS2 PCR and sequencing primers:
G2 fwd primer: 5'- GAGCTGGGAAGGGGATCCTG **-** 3'
G2 rev primer: 5'- GGGACCAGGAGCGGGACC **-** 3'

Figure S2

Off-target evaluation of target sites for PPP1R12C:

	Guide Sequence + PAM	Specificity Score	Predicted Efficiency		Out-of-Frame Score	Total/in-exon off-targets	in-exon off-targets (# of mis-matches; gene)
			Doench '16	Mor.-Mateos			
**C2:**	AGGGAGACATCCGTCGGAGA AGG	95	52	52	75	62/7	4:exon:AC096574.4<br>4:exon:AC096574.4<br>4:exon:HBP1<br>4:exon:LSM14B<br>4:exon:COL20A1<br>4:exon:BCAS3<br>4:exon:MAML3
**C3:**	CACGTGGTGCAGCGCCGAGA AGG	92	49	67	71	58/13	4:exon:RP11-862L9.3/MBP<br>4:exon:SHANK1<br>4:exon:TEN1-CDK3/TEN1/CDK3<br>4:exon:RERE<br>3:exon:RP11-161M6.2/LMF1/AC009041.2<br>4:exon:COMP<br>3:exon:ADAM11<br>4:exon:PDCD2<br>4:exon:CDK16<br>4:exon:ICOSLG<br>4:exon:CTSB<br>4:exon:PCDP1<br>4:exon:RASIP1
**C4:**	TGGGGGTTAGACCCAATATC AGG	89	41	50	67	65/2	4:exon:RGS14<br>4:exon:UBE2L6
**T2:**	GGGGCCACTAGGGACAGGAT TGG	61	59	54	68	247/22	4:exon:DAK<br>4:exon:AMN/CDC42BPB/RP11-365N19.2<br>4:exon:LINC00094/BRD3<br>4:exon:AEN<br>4:exon:FUT1<br>4:exon:PPP1R37<br>4:exon:NOS2P3<br>4:exon:GPR37L1/ARL8A<br>4:exon:CSRP1<br>4:exon:NPPA/NPPA-AS1<br>4:exon:SOGA1<br>4:exon:RNF4<br>4:exon:SCAF1<br>4:exon:METRN<br>3:exon:C20orf112<br>4:exon:DNAH1<br>4:exon:TGFB1I1<br>4:exon:SRCIN1<br>4:exon:DHX38<br>4:exon:CSK<br>4:exon:COL18A1/SLC19A1<br>4:exon:ALOXE3
